# Exploring perceptions and experiences of stigma in Canada during the COVID-19 pandemic: a qualitative study

**DOI:** 10.1186/s44263-023-00020-7

**Published:** 2023-12-02

**Authors:** Jeanette Cooper, Suvabna Theivendrampillai, Taehoon (Tom) Lee, Christine Marquez, Michelle Wai Ki Lau, Sharon E. Straus, Christine Fahim, in partnership with the Chinese Canadian National Council Toronto Chapter, the Chinese Canadian National Council for Social Justice

**Affiliations:** 1 Knowledge Translation Program, Li Ka Shing Knowledge Institute, Unity Health Toronto, 209 Victoria Street, Toronto, M5B 1T8 Canada; 2 Chinese Canadian National Council Toronto Chapter, 105-1911 Kennedy Road, Scarborough, Canada; 3 Chinese Canadian National Council for Social Justice, 505-123 Edward Street, Toronto, Canada

**Keywords:** Stigma, East Asian, Chinese, Canadian, Racism, COVID-19, Qualitative research, HSDF

## Abstract

**Background:**

The COVID-19 pandemic fueled stigmatization and discrimination, particularly towards individuals of Chinese or East Asian ethnicity. We conducted interviews with members of the public in Canada in order to describe and understand stigma perceptions and experiences during the COVID-19 pandemic.

**Methods:**

We used a phenomenological approach to describe stigma experiences of Canadian residents during the COVID-19 pandemic and compared the stigma perceptions and experiences of East Asian and non-East Asian individuals. Participants were invited to take part in a single, semi-structured interview. The interview guide was rooted in the Health Stigma and Discrimination Framework (HSDF). Interviews were conducted in English, Mandarin, and Cantonese. Following participant consent, interviews were audio recorded and transcribed verbatim. Data were double coded and analyzed using qualitative content analysis guided by a framework approach.

**Results:**

A total of 55 interviews were conducted between May and December 2020. Fifty-five percent of the sample identified as East Asian, 67.3% identified as women, and mean age was 52 years (range 20–76). Fear of infection, fear of social and economic ramifications, and blame for COVID-19 were reported drivers of stigma. Participants described preexisting perceptions on cultural norms and media influence as facilitators of stigma that propagated harmful stereotypes, particularly against Chinese and East Asian individuals. Participants observed or experienced stigmatization towards place of residence, race/ethnicity, culture, language, occupation, and age. Stigma manifestations present in the public and media had direct negative impacts on East Asian, particularly Chinese, participants, regardless of whether or not they personally experienced discrimination.

**Conclusions:**

We used the HSDF as a rooting framework to describe perceptions and impact of stigma, particularly as they related to race/ethnicity-based stigmatization in Canada. Participants reported a number of drivers and facilitators of stigma that impacted perceptions and experiences. These findings should be used to develop sustained strategies to mitigate stigma during public health emergencies or other major crises.

**Supplementary Information:**

The online version contains supplementary material available at 10.1186/s44263-023-00020-7.

## Background

The COVID-19 pandemic fueled increased stigmatization towards those perceived to be of East Asian ethnicity in Canada. People of Chinese and East Asian ethnicity were blamed for the pandemic, and media channels widely referred to the COVID-19 pandemic as the “China virus” [1]. International calls to ban Chinese nationals circulated via social media platforms and online petitions [2]. With this came a sharp rise in discrimination and racist attacks towards people of East Asian descent in public and workplace settings [3].

Similar patterns were observed in prior epidemics, including the 2003 severe acute respiratory syndrome (SARS) outbreak in Toronto. Media reports on SARS-labeled SARS as the “Chinese disease” [4] and concerns about the SARS outbreak were tied more broadly into concerns about increasing globalization and multiculturalism in urban cities [4–7]. The result was amplification of racist and anti-Asian rhetoric, resulting in racialization (a term [8] to describe individuals who may be perceived as racially different than the racial or ethnic majority) of Chinese and East Asian populations and direct impact on their experiences in healthcare, business, and travel.

Stigma, misinformation, and fear during an infectious disease outbreak are often manifestations of broader organizational, community, and historical systems, cultures, and beliefs that shape the public response. The Health Stigma and Discrimination Framework (HSDF) describes the health stigmatization process through this intersectional lens. Unlike other stigma frameworks, the HSDF provides a broader understanding of health-related stigma within a socioecological context and considers how intersecting characteristics such as race, gender, and age interact with social and structural contexts to manifest stigma [9].

The purpose of this study was to use the HSDF to guide interviews with members of the public in Canada in order to describe and understand stigma perceptions and experiences during the COVID-19 pandemic. Guided by integrated knowledge translation methodology, which is the process of working with knowledge users to design, conduct, and implement research, we partnered with Chinese-Canadian advocacy organizations to design and conduct this study. In particular, we aimed to describe the stigma perceptions towards and experiences of Chinese and East Asian residents of Canada during the COVID-19 pandemic.

## Methods

We report our methods and findings in accordance with the consolidated criteria for reporting qualitative studies (COREQ, Additional file 1) [10].

### Research team and reflexivity

Interviews were conducted by research staff from the Knowledge Translation Program (J. C., C. M., T. L., M. K. L.). Interviewers held a bachelor or master’s level degree and were experienced in qualitative methodology. Three interviewers were women and one was a man. Interviewers were diverse in race/ethnicity and included individuals of East Asian ethnicity; M. K. L. conducted the Cantonese- and Mandarin-language interviews. Interview data were transcribed and translated to English by the Chinese Canadian National Council Toronto Chapter. We aimed to mitigate potential biases in interviewing and analysis by having predefined questions and prompts to limit scope creep and support a standardized interview across participants. We also participated in reflection exercises on potential biases and how they might impact the study and interpretation. The interviewers did not hold any relationships with study participants. Participants were aware that researchers were conducting the study in collaboration with Chinese-Canadian advocacy organizations and that the study questions would center on stigma during the COVID-19 pandemic.

### Study design

This study was rooted in a phenomenological approach [11] — the intention was to understand the stigma experiences of residents of Canada during the pandemic, and particularly, to compare the stigma experiences and perceptions of Chinese/East Asian to non-Chinese/East Asian residents. We used purposive and snowball sampling methods to recruit a population that was diverse in race/ethnicity and age. We aimed to conduct a minimum of 50 interviews yet planned to continue interviewing until saturation was reached. Recruitment materials and interviews were offered in English, French, Cantonese, and Mandarin. Recruitment flyers and email invitations were distributed through social media (i.e., Twitter, Kijiji), email, and newsletters distributed by academic (e.g., Strategy for Patient-Oriented Research Evidence Alliance), hospital (e.g., St. Michael’s Hospital), and community advocacy organizations (e.g., Chinese Canadian National Council Toronto Chapter and Chinese Canadian National Council for Social Justice). Interested participants completed an online registration form and were then contacted by a study staff member (T. L., research assistant, BSc). Study staff invited participants to complete a demographic screening questionnaire (which gathered information on participants’ age, race/ethnicity, and occupation), hosted on the Qualtrics platform [12]. Participants who worked in a health or public-policy field were invited to participate in a complementary research study that explored the perceptions of stigma among health professionals and policy-makers, respectively. We then used the demographic questionnaires to form a sample that was diverse in race/ethnicity (aiming for equal representation of Chinese/East Asian and non-Chinese/East Asian participants) and diversity in age. Selected participants were invited to schedule an interview in their preferred available language. Participants who completed an interview were also invited to provide additional demographic data (in the form of an online questionnaire or verbally, during their interview) related to employment status, sex, gender, and education.

An interview guide (Additional file 2) rooted in the HSDF [9] was developed. This guide contained 22 questions related to the participant’s perceptions of COVID-19 stigma drivers (the negative factors that give rise to stigmatization), stigma facilitators (the influencing factors that can amplify or curtail stigma), and stigma marking (stigmatization of a certain group of people because of a certain health condition or demographic, socioeconomic, or other factor) [9], as well as their experiences with stigma and recommendations on strategies to address pandemic-related stigma. To minimize leading participants, we did not probe for perceptions related to Chinese or East Asian stigmatization; rather, we posed open-ended questions to ascertain participants’ perceptions of who was being stigmatized during COVID-19 and how.

Verbal consent for the interview and the recording was obtained by the interviewer before the interview began. Interviews were conducted in the language preferred by the participant through the St. Michael’s Hospital secure teleconference line and were audio recorded. Participants were informed that only the interviewers were present on the teleconference line. Participants took part in a single interview; no member checking was conducted. None of our study participants preferred to complete their interview in French. Participants were provided with a CAN $25.00 honorarium post-participation.

### Data analysis

The English interview audio recordings were transcribed through NVivo Transcription [13] and were de-identified and reviewed for accuracy by a study staff member (T. L., C. M., and S. T.). The Mandarin and Cantonese interview audio recordings were de-identified by the study team, transcribed by our community partner, the Chinese Canadian National Council — Toronto Chapter (CCNCTO) and were reviewed for cultural interpretation and accuracy by a study staff member (M. K. L.).

All interviews were analyzed using qualitative content analysis guided by a framework approach [14]. A coding framework was developed a priori, guided by the study objectives and the HSDF (Additional file 3). All interviews were double coded by two study staff members (T.L, C.M., J.C., S.T.). Discrepancies between coders were discussed until a kappa > 0.6 (indicating moderate agreement) was reached. Once coding was complete, interview data were charted into a framework matrix, and themes were developed by study staff members from the coded data and transposed to the domains of the HSDF [9].

## Results

### Demographics

Fifty-five interviews were conducted between May and December 2020. Twenty-nine interviews were conducted in English, 17 in Mandarin, and 9 in Cantonese. The mean interview length was 58.16 min. The median age for the sample was 52 years (range 20–76). The majority of participants were women (*n* = 37, 67.3%). Fifty-five percent of the sample was East Asian (*n* = 30), and the majority held a university degree (*n* = 44, 80%). The majority of participants resided in the province of Ontario, Canada (*n* = 43, 78%) (Table 1). The final kappa values for coding were 0.91 for both the English and Mandarin/Cantonese interviews, indicating very high levels of coder agreement.

**Table 1:** Demographics

Demographics	Number of individuals (*n* = 55)
**Gender**^a^	
Male	17 (*EN* 18.0%, *CN* 13%)
Female	37 (*EN* 33%, *CN* 35%)
Unanswered	1 (2%)
**Age**	
18–34 years	16 (*EN* 20%, *CN* 9%)
35–54 years	18 (*EN* 15%, *CN* 18%)
55 + years	19 (*EN* 15%, *CN* 20%)
Unanswered	2 (4%)
**Ethnicity (multiple selections possible)**	
East Asian	30 (*EN* 7%, *CN* 47%)
White/Caucasian-North American	10 (*EN* 18%)
White/Caucasian-European	9 (*EN* 16%)
Other (Black-Caribbean, Latinx, South Asian, Southeast Asian, Middle Eastern)	6 (*EN* 11%)
Unanswered	2 (4%)
**Completed education**	
Graduated high school or equivalent	8 (*EN* 5%, *CN* 9%)
Postsecondary degree	26 (*EN* 24%, *CN* 24%)
Postgraduate or professional degree	19 (*EN* 20%, *CN* 15%)
Blank	2 (4%)
**Province/territory**	
Western Canada (BC, AB, SK, or MB)	7 (*EN* 13%)
Eastern Canada (ON, QB, NS, NB, PEI, NL)	47 (*EN* 32%, *CN* 55%)
**Region**	
Large urban population center, with a population of 100,000 or more	40 (*EN* 31%, *CN* 42%)
Medium population center, with a population between 30,000 and 99,999	8 (*EN* 9%, *CN* 5%)
Small population center or rural area, with a population up to 29,999	7 (*EN* 13%)
**Employment status**	
Full time	20 (*EN* 20%, *CN* 16%)
Part time	6 (*EN* 4%, *CN* 7%)
Self-employed or student	5 (*EN* 5%, *CN* 4%)
Not employed	9 (*EN* 5%, *CN* 11%)
Retired	13 (*EN* 15%, *CN* 9%)
No response	2 (4%)
**Kappa value**	
English interviews	91.03
Chinese interviews	90.91

^a^Participants in our sample identified as “female gender” or “male gender.” None of our study participants identified as nonbinary. EN and CN denote the proportion of the sample who completed their interviews in English or Mandarin and Cantonese, respectively

#### Theme 1: Fears of COVID-19 infection and pandemic impacts were widespread. Chinese participants contended with the fear of experiencing racism due to COVID-19 stigma

COVID-19 fears were widespread. Participants feared becoming sick with COVID-19 or having long-term health consequences related to infection. Participants also feared the disruption of normal activities, such as work or school attendance. These fears often impacted their behaviors and daily activities, as demonstrated in the following quote:

I’m worried about getting it and that’s why I stopped getting my newspaper so I wouldn’t have to touch something that someone has already touched. I’m worried about touching parcels without waiting or cleaning them up… It’s probably paranoid because supposedly it doesn’t last on paper but I have been pretty worried about that - P64, Female, White - European

Participants worried about the impact COVID-19 could have on their personal finances, including effects of the pandemic on the economy and the possibility of job loss or difficulty finding a job due to COVID-19, as seen in the following participant quote:

Well, from a household perspective, my fiancée is self-employed and this has decimated his business that we had been building up for two years on already. Financial stress is definitely high on the list – P307, Female, White – North American

Many of these fears were compounded for those of Chinese ethnicity who felt a heightened fear that they would also have to contend with racial discrimination amidst these public fears, as demonstrated in the following quotes:

Many of us don’t speak English very well…I see Chinese get scolded on the streets because they walked too close and touched someone carelessly. They got scolded for being Chinese and people even swear at them. The Chinese were very frustrated and didn’t know what to say back and could only walk away. Honestly, many Chinese have had such experiences…spitting is scary, because of COVID-19; they might infect you if they have COVID-19.- P78, Female, East Asian

If I get the virus, I won’t be able to find a job, and as a Chinese [person], there may be racial discrimination against me. I will also be isolated due to the virus – P365, Male, East Asian

#### Theme 2: COVID-19 fears heightened stigmatization towards those perceived as having, or being at higher risk for, COVID-19. Often, this stigma and blame was directed towards Chinese or East Asian individuals but was also directed towards those perceived to be “bringing in” COVID cases or those not helping to mitigate COVID-19 spread

Most participants perceived that people of Chinese ethnicity (or those who appeared to be of Chinese ethnicity) were blamed for COVID-19 in Canada. Participants believed that Canadians perceived those of Chinese ethnicity as carriers of the virus or at fault for the virus emerging in the human population, as seen in the following quote:

Now that [COVID-19] has become such a big deal, many people are affected, they have lost their jobs or become ill. People must be very angry so the simplest way to vent is to blame the Chinese. Many of them are scared, or have been affected negatively, so who can they blame? Of course they will blame those who are easiest to blame – P387, Female, East Asian

Others, particularly non-East Asian participants, blamed the government of China as responsible for COVID-19 while recognizing the negative impact this blame had on individuals of Chinese ethnicity, whom they felt were unfairly targeted:

Yeah, essentially the problem is that people are blaming some average Joe, average Jane, for all of this. They're not blaming the people responsible, the government. When the whole pandemic started in China, it was the wet markets in Wuhan and talk of the controversial labs and people saying that's where it came from. I think people are saying, oh, those people there, they eat all the bad stuff in the wet market so those Chinese people - that have never even been to China, or anything - in Canada, oh they [Chinese people] must do the same thing... They are generaliz[ing], on the actions of what people over there do, even though they don't do that here. – P996, Male, White – North American and Middle Eastern

These sentiments were often embedded in racist or stigmatizing perceptions related to Chinese culture or cuisine, as identified in the following quote:

Yes, unfortunately for Asians in general, there is perhaps the assumption that – you Asians are all the same and because you have wet markets and because of your consumption is the reason why this virus has proliferated and started in the whole place – P106, Female, Ethnicity not specified

Non-East Asian participants also feared those who were “bringing in” COVID-19; this sentiment was directed towards ‘travellers’ (in this context, meaning those with non-Ontario -e.g., other provincial/territorial or US- license plates), as seen in the following quotes:

Their dual citizenship and their cars have American plates on them. People assume that somehow they’re American, that sort of snuck across the border and shouldn’t be here and their cars have been keyed, scratched – P63, Female, White – North American

There was a case last week where a note was left on someone’s windshield when they were at a beach for a walk and they came back and had a note that was incredibly rude and it had like the F word, like F off back to Nova Scotia because they had Nova Scotia plates – P835, Female, White - European

The stigma that Americans are being given by Canadians and not wanting them to cross the border and for the most part, Canadians wanting that border to stay closed…so again, the stigma of ‘you may have it, and we don’t, and don’t bring it to us’. – P507, Female, Ethnicity not specified 

#### Theme 3: COVID-19 fears uncovered underlying, racist beliefs in Canada towards people of Chinese ethnicity

Many East Asian participants described the negative and racist attitudes towards Asian communities that existed in Canada prior to the pandemic. They felt that COVID-19 provided fodder for individuals to behave in a discriminatory or stigmatizing manner and drive racist narratives, as identified in the following quotes:

I am most worried about the people who have other motives, or those who already dislike Asians for other reasons. They may not even really believe that Chinese people or Asians brought COVID-19 here. They’re only using COVID-19 as an excuse to attack the Asian community. P47, Female, East Asian

Actually even before COVID-19, someone has asked me ‘Why are you Chinese so rich?’ and I said ‘No I’m normal, I don’t have money’. Then they say ‘No, you Chinese are very rich. See the condos around here are all bought by Chinese’…what they mean is that Chinese will buy many properties and raise the prices, and then even the high-income locals can’t afford to buy property. Some of them say this – ‘if you Chinese keep doing this then we won’t welcome you’. When I hear that, I get scared you know, but I can only smile…when they see a Chinese [person], they would think you’re rich and you created this inflation so of course they hate you. On top of that, now there’s COVID-19 that caused them to lose their jobs, they dislike us even more – P78, Female, East Asian

Chinese people have become the scapegoat…it feels like all the other races are blaming Asians and Chinese for bringing the virus here. It feels like they want us to leave Canada. This is not fair. The racial discrimination is very serious. People don’t focus their attention on how to overcome this virus and attack other people instead. This type of atmosphere is really bad – P849, Female, East Asian

Non-East Asian participants also noted similar themes:

It angered me when I started to hear rumors that Dr. Tam, because she’s Asian, might be in cahoots with China and wasn’t really on the side of Canadians. Like who thinks up this stuff? Give me a break! I’m glad that seemed to disappear fairly quickly – P63, Female, White – North American

#### Theme 4: Participants felt that Canadian and United States (US) media coverage of COVID-19 facilitated stigmatization of Chinese communities and perpetuated harmful perceptions about cultural norms

Most participants felt that the media’s politicization of public health, coverage of pandemic restrictions, and coverage of stigmatizing comments from influential politicians further led to discrimination against those perceived to be of East Asian ethnicity. Some participants gave examples of the extensive media coverage of US President Donald Trump’s comments where he referred to COVID-19 as the “Wuhan virus” or the “China virus,” as seen in the following quotes:

There’s the influence of the President of the U.S., Trump, who keeps saying the virus is the ‘Kungflu virus’ or ‘the Chinese virus’. He is making things worse. He is the US president, so many people in the US and Canada believe what he says…His words carry weight… Nevertheless, Chinese and Asians are stigmatized. This is very unfair and sinister. – P849, Female, East Asian

On the internet, many people say this is the China virus, or the Wuhan virus. However, the virus knows no borders. To name it that way is racist and it will lead to discrimination against overseas Chinese here. That makes me afraid – P510, Female, East Asian

A non-East Asian participant noted that media stories also depicted other racialized groups negatively, as demonstrated in the following quote:

P: It was obviously younger people of a certain ethnic race that were having these backyard parties daily and that apparently they were spreading the virus unknowingly…and for the mayor to come out and basically say that we are cracking down on these social gatherings…there was like five or six of them in the picture and they were just standing close together and they’re laughing, all that stuff. And that’s the picture they put out in the media. So it kind of gives you that perspective of when it could be any other cultural race – even I heard of in my general area of older people, 50 plus, they’re white, Caucasian, still having large gatherings

I: The picture of the young individuals staying together that was posted, were there specific ethnicities that were targeted in those pictures?

P: It looked like they were Indian - P762, Male, White - European

#### Theme 5: Intersecting stigma drivers (fear, blame) and facilitators (perceptions of cultural norms, media influence) led to stigmatization of individuals based on multiple intersecting demographic factors

Participants most commonly reported stigmatization based on race/ethnicity and language but also described stigma related to age and occupation. Often, these stigmas intersected, leading to compounding effects. Participants of East Asian and non-East Asian ethnicity most commonly described racialized stigma directed towards people of Chinese ethnicity, as demonstrated in the following quotes:

Yes, some Asian people have been targeted…I think people want to find someone to blame and China is so darn far away so what becomes a target is someone who has nothing to do with China, maybe 3^rd^ of 4^th^ generation Canadian but because they are a visible minority they have been unfairly blamed. It’s through ignorance, people want somebody to blame – P64, Female, White - European

I think almost everyone takes for granted [or assumes] that Chinese people eating bats is what caused COVID-19. That’s very bad for Chinese people. Even when I go out, I hear people say that. But I can’t do anything – P78, Female, East Asian

This stigmatization was rooted in the aforementioned stigma drivers related to fear of the pandemic, blame towards China and Chinese persons, and amplified by stigma facilitators related to underlying systemic racism towards persons of Chinese ethnicity and perceptions of cultural norms driven by media narratives.

In particular, one of the most salient themes uncovered in our research was related to the intersecting effect of Chinese/East Asian ethnicity and masking. Many participants perceived differences in the cultural significance of mask wearing in North America as compared to East Asia. According to participants, masking in East Asian cultures is seen as a preventive health measure, while North American culture perceives masking as an indicator that the mask wearer is ill. Participants believed these perceptions led to stigmatization of East Asians in Canada, particularly early in the pandemic prior to mask mandates, as demonstrated in the following participant quotes:

I cannot understand why Westerners regard mask-wearing as a signal of being sick. To us Chinese, wearing a mask is also a way of protecting ourselves. Chinese people have experienced discrimination because of this, although everyone is now required to wear a mask. – P397, Female, East Asian

At my workplace, there was one time when an Asian colleague wore a mask. The boss asked him why he needed to wear a mask and he replied, ‘no, no, no, I don’t have COVID, I just have a cough that’s why I’m wearing a mask’. People think Asians are more susceptible to this disease…these are examples of discrimination – P701, Female, East Asian

First there was an outbreak in China, so many Chinese people here followed the practices in China and started to wear masks…Westerners looked at us Chinese who wore masks differently – P701, Female, East Asian

I saw this Chinese woman got harassed on the bus when I was going home one night. They said, she was wearing a mask and people were saying, ‘oh, are you wearing a mask?’ and they were mocking her because they thought she was spreading the virus – P516, Male, Black - Caribbean

Stigmatization based on race/ethnicity was most widely reported towards Chinese individuals. However, both East Asian and non-East Asian participants also reported perceptions of race-based stigma during the COVID-19 pandemic directed towards other racialized groups, including Indigenous and Black populations, American citizens, and people of Italian ethnicity, who were perceived to be at higher risk for COVID-19:

This virus really has made people suspect one another. For example, when COVID-19 was very serious in Italy, my family and I would avoid going to Italian restaurants…because they may have a higher chance of being carriers of the virus. Of course I understood later, that COVID happening there doesn’t mean those people are virus carriers. It’s a very weird feeling – where COVID happens we would be suspicious of people from those places – not suspicious, but we would try to keep a distance from those people. – P252, Female, East Asian

Well, one of the Black communities here had been seriously affected because many care workers came from…Haiti, from the United States…some of the people went to the [long term] care homes...then this was the people sometimes got infected and then there were outbreaks in their communities. I think that I said, you know, I hate to say this, but I feel so bad, but now if I see a Black person, I’m anxious because maybe I think they’ve had a greater risk of being exposed, and that’s really ugly and not good at all. –745, Female, White – North American

I think the Black community too has been stigmatized…I do feel like people have been stigmatized, definitely racial groups. Definitely. – P573, Female, White - European

Stigma marking during the COVID-19 pandemic was complex. In the following quote, we see an example of the interplay of stigma manifestation, coupled with fear as a stigma driver. In the quote, the participant describes her experience of being stigmatized because she was a Chinese person wearing a mask; yet, the participant’s fears are also rooted in a stigma that the person making the comments might have COVID-19 because they are not wearing a mask and therefore must not have “hygiene awareness”:

I remember taking the subway and walking on the streets, maybe because they saw me wearing a mask… and maybe they didn’t know if I was Chinese or not because I was wearing a mask, but they would say things like, “You have the virus. You’re sick. You should stay away from me.” I felt very frustrated/helpless, but because they weren’t wearing a mask, I couldn’t argue with them or explain to them. What if they have COVID-19? What if I get COVID-19 from them by talking to them? They don’t have hygiene awareness, but there’s nothing I can do about it- P78, Female, East Asian

In another quote, a participant describes the complexity of recognizing the harm of race-based stigma, while noting similar narratives within her Chinese community:   

Even within my own family, we do talk about it candidly and even the perception of Chinese people, by Chinese people, is also quite, quite staggering, because even my father sort of holds those views too. When this whole thing started, he was like, maybe we shouldn’t go to [a specific grocery store] because there’s a lot of Chinese people – P009, Female, East Asian

Non-East Asian participants perceived that those of Chinese and other racialized ethnicities, younger and older age, and occupation (including frontline workers, nurses, immigrant populations, and those working in agriculture) were also stigmatized during the pandemic, as demonstrated in the following quotes:

I have to say that one stigma that I didn’t mention, that’s pretty apparent these days, is age. In two ways, age because an older person in the home already…it’s so bad, its almost like they don’t want to put the money there [in long-term care homes], so that’s a little bit of discrimination…[and] like some older people may be more stigmatizing the younger people saying, ‘well, I mean, look at Trinity Bellwood Park, no one was there under 30 sitting around the park’ – P328, Female, South Asian and White - European

Well, I don't know if you've heard of it. There was the “boomer remover” memes that was floating around when this first started. People saying, oh, it just affects the boomer and they were talking about how it affects them predominately to the point that, the young people don't have to care and people my age don't have to care. No one but the elderly people have to care. I think a lot of old people are being completely shafted too, because they didn't do anything wrong. They just happen to get old… COVID hits and now they're saying old people should just drop dead. I don't believe that. I don't believe old people should die just because they happened to be old. They should be able to live their lives in peace and comfort as much as possible. And I think a lot of times people, I think all COVID has done is just giving groups ammunition to attack the people they always hated – P996, Male, White – European and Middle Eastern

I've heard cases where nurses and frontline workers haven't been, even doctors haven't been allowed to go into a bank because people are afraid of getting it [COVID-19]. So these people are fighting for everybody and they're not letting them have essential services. That's just not right – P573, Female, White - European

For those with intersecting characteristics that were stigmatized, the effect of COVID-19 stigmatization was compounded. This experience is summarized in the following quotes from Chinese participants:

In general, being a visible minority, and a woman, I think people of colour in general have this heightened awareness to represent and just sort of always be ‘on’ when interacting with other people. But I think this COVID-19 has certainly very much heightened that. Every action I take, I’m just very aware that that could be the one experience that others remember Chinese people for – P009, Female, East Asian

For instance, consider my friend who works at the long-term care home. He has underlying diseases so he decided not to work after the outbreak of COVID-19. Some people said he was behaving like an irresponsible coward, that Asians like him were cowardly – P531, Female, East Asian

#### Theme 6: Most participants did not personally experience discrimination or stigmatization during the pandemic; however, almost all participants were aware of instances of COVID-19 stigmatization due to race/ethnicity, age, occupation, or a combination of these factors

Though few participants reported personally experiencing discrimination or stigmatization, most participants cited known instances of stigmatization against people who appeared to be of Chinese ethnicity, which they witnessed personally or heard of through media reports. These instances included verbal attacks (including the use of racial slurs), graffiti painted on businesses, strange looks being given in public spaces and physical attacks, and impacts on businesses, as noted below:

I haven’t encountered problems in my work because I’m Chinese…but I saw on social media that some people in the service industry were verbally attacked at work by non-Chinese because they were Chinese. COVID-19 is not their fault but even so they were suddenly verbally attacked and called bad names just because they were Asian. – P104, Female, East Asian

Some participants noted instances of physical attacks:

I haven’t experienced it [stigmatization] personally, but I know Chinese and even Asians have been targeted…some Chinese stores and restaurants have been boycotted or even forced to close down. Walking down the street or in supermarkets, Chinese people may be verbally or even physically attacked. They may experience both physical and mental harm. Stigmatization should be condemned. – P883, Female, East Asian

I do, I think that a lot of people have blamed China for bringing the virus here …there was a boy here that got beat up here in Saskatoon, because they think it was because of some racism because he was Asian, but he wasn’t from China – P707, Female, White – North American

Some participants perceived that COVID-19 stigmatization was an infrequent problem in Canada and was more prevalent problem in other countries such as the US. These participants also felt that stigmatization decreased over the course of the pandemic, particularly as COVID-19 became prevalent globally.

#### Theme 7: Some East Asian participants personally experienced  stigmatization during COVID-19. These participants felt uncomfortable navigating public spaces and worried about their safety and public perceptions of their ethnic group. Others described negative impacts on mental health

Participants who personally experienced stigmatization described being the targets of strange looks or unkind comments in public spaces. They attributed these stigmatizing actions to a few factors including the following: experiencing a possible symptom of COVID-19 (e.g., coughing), being perceived as not in compliance with public health measures, using PPE (e.g., masks) in a public setting before this was commonplace, speaking a different language or experiencing a language barrier, and their race, as demonstrated in the following quotes:

I think because of my background, I knew about COVID-19 earlier than Canadians. I think Canada’s health measures were delayed…most people didn’t know about the virus back then, so they thought it was strange that I was wearing a mask. Now that the government tells people to wear masks, of course it’s not strange anymore, so people don’t give me weird looks. I haven’t encountered any discrimination or dramatic events, but in the beginning people would look at me strangely – P534, Female, East Asian

My experience was that White people would look at me with disgust if I wore a mask while shopping, but I would not be treated like this for wearing a mask in Chinatown- P295, Female, East Asian

This stigmatization led to East Asian participants having feelings of self-consciousness, and hesitancy in navigating public spaces out of fear, as demonstrated in the following quotes:

[COVID-19] has brought fear to the Chinese community. Why do I say this? I am concerned that there will be discrimination against Chinese because of the virus. People will think that Chinese people are not good and that Chinese people spread the virus all over the world – P510, Female, East Asian

Whenever I go out for groceries and such I'm kind of hyper aware that because I am Chinese that this sort of heightened responsibility and pressure that I feel to not give other people reason to think that this is the fault of Chinese people. So things like wearing a nonmedical mask and even when, for example, when I’m at Costco, I'm hyper aware of what I touch and if other people will see me touch things and then not buy it. So I just think that there's sort of this heightened level of awareness that I have now. And I think in part that's because of the color of my skin. – P009, Female, East Asian

We are all very careful…we experienced SARS so we know how to avoid those situations. We won’t do things that would lead to discrimination against or us things that would be perceived by others as risky. So we stay home most of the time, not going anywhere. And we don’t shop at Westerners’ stores…the biggest impact is that our activities are now limited – P120, Male, East Asian

Some participants described in detail the tremendous impact that intersecting stigmas had on mental health:

Asian women or Chinese women in particular may be attacked and abused and experience discrimination. They may feel like they are suffering a lot. In addition to the stress of the pandemic, they may experience double suffering due to stigma, discrimination, attacks and even humiliation – P849, Female, East Asian

It has had a huge negative impact on the overseas Chinese population in particular. There’s a psychological impact…these days, when I go out – and maybe its psychological – I feel like other people are thinking ‘Oh, Chinese’, when they look at me. As soon as I hear people say the word ‘Chinese’, I feel uneasy…I didn’t feel this way before the pandemic. Now, I worry that we have to take some responsibility for the virus. I feel ashamed. –P510, Female, East Asian

#### Theme 8: Participants, particularly those of Chinese ethnicity, highlighted the need for advocacy against Asian racism

Many participants felt the Canadian government did not “do enough” to stop anti-Asian rhetoric during the COVID-19 pandemic, as demonstrated in the following quotes:

The [Canadian] government failed to clearly explain to the public the origin and cause of the disease, which led to a lot of fear among the public. The government also failed to give enough attention to the disease. Stigmatization is the result of this lack of transparency and unclear information – P120, Male, East Asian

It is understandable that people who are not familiar with China or don’t have associations with China will think that it is what happened in China that caused what’s happening here…I think the Canadian government hasn’t done anything in particular to help or worsen the situation regarding blame – P104, Female, East Asian

East Asian and non-East Asian participants highlighted the importance of advocacy against racism and stigmatization due to COVID-19:

I think the community should do more events or promotion. Like Black Lives Matter. We should let people know that Chinese people deserve respect too. We shouldn’t be treated like that or bullied just because we’re Chinese - P78, Female, East Asian

We can’t let [stigmatization] block us, or our legitimate rights and interests may not be protected. If we experience stigma, we should focus on what we should do to fight against it – P531, Female, East Asian

I heard a couple of times the Prime Minister talking about this issue [of racism against Dr. Teresa Tam, chief medical officer]…I think it’s important for the leaders to show that this is racist, it’s not good sense. Mention it as much as possible – P451, Male, White - European

## Discussion

**Fig. 1:**
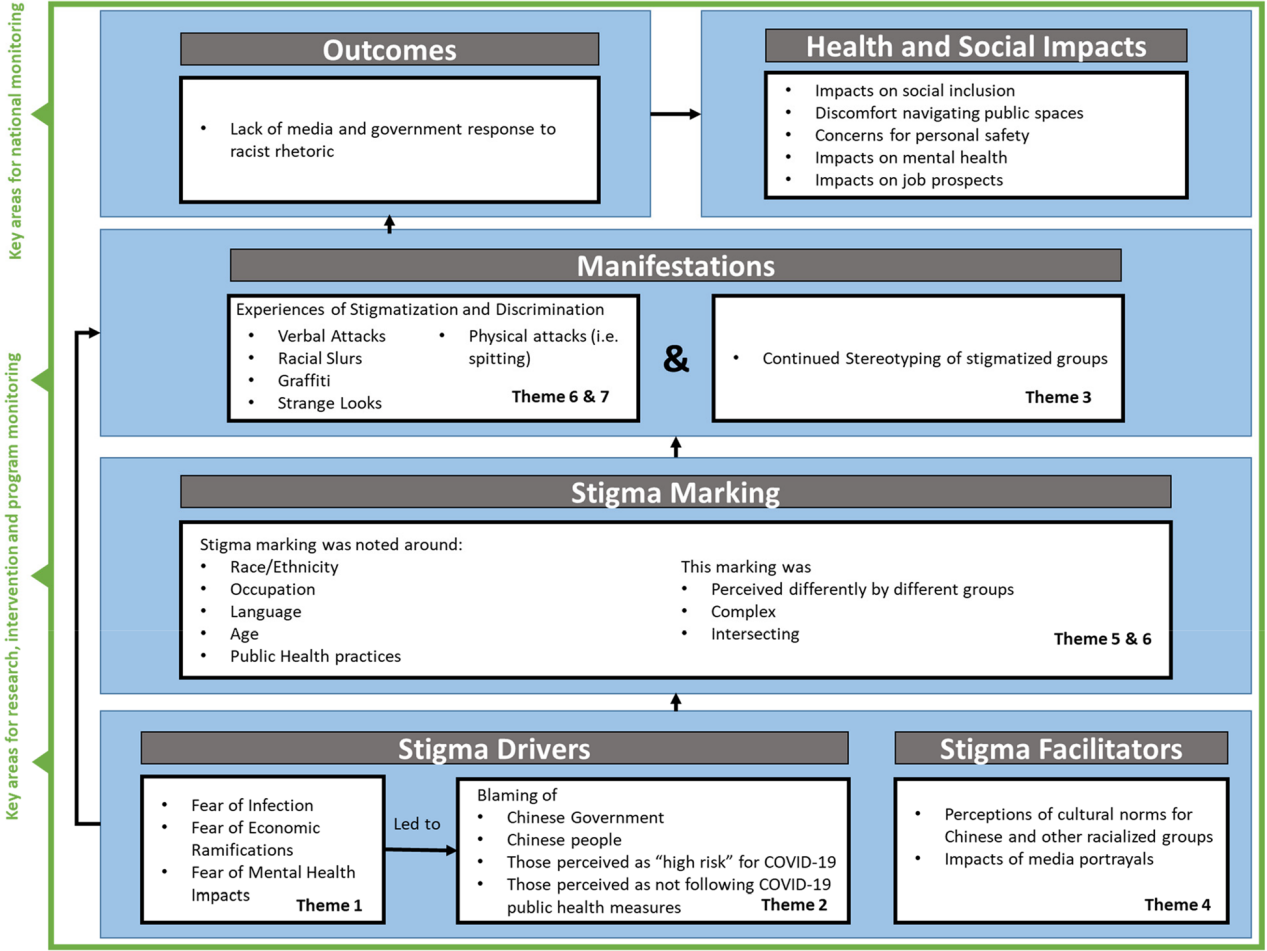
Summary of study findings, categorized using the HSDF

We used the HSDF to describe perceptions of stigmatization in Canada during COVID-19. Figure 1 depicts our findings of how stigma drivers and facilitators led to stigma marking and manifestations. In our study, fear was the rooting stigma driver, which is consistent with published literature showing fears and concerns as the dominant emotions during the early stages of the pandemic [9, 15, 16]. Stigma drivers related to perceptions of cultural norms and media portrayals reinforced stigmatizing beliefs and attitudes, particularly towards those of Chinese or East Asian ethnicity [15–20]. These stigma drivers amplified racist and negative stereotypes that predated the pandemic.

Canada has a long history of excluding Asian populations (e.g., the Chinese Head Tax and Exclusion Act [21]), further driving the concept of the “perpetual foreigner,” where Asian Canadians (including those born in Canada or who have lived here for generations) are perceived as foreigners who are loyal or deeply connected to China. Anti-Asian sentiments were not unique to the COVID-19 pandemic and have proliferated during previous public health emergencies in Canada, including the 2003 SARS outbreak and the 2009 H1N1 outbreak [22, 23]. This was noted by our study participants who perceived being “othered” and treated as foreigners in Canada, including by participants who experienced similar discrimination during the SARS outbreak.

These findings align with a survey that we conducted with Ontario residents between June and December 2020. Fifty-one percent of our sample (*n* = 1823) felt that racist views had increased in Canada during the pandemic, and 37% felt this stigma was based on race/ethnicity [17]. Non-White participants were more likely to fear experiencing stigma, and East/Southeast Asian participants were 18.7 times more likely to fear stigma or racism compared to White participants. This mirrors emerging data from the US, including a research study showing that 23% of Asian individuals sampled personally encountered pandemic-related stigmatization, including in the workplace [24].

Stigma marking was complex, intersecting, and perceived differently by different participant groups. For instance, many East Asian participants connected COVID-19 stigma experiences with their race and use of masks, while non-East Asian participants attributed COVID-19 stigma to other factors including occupation, age, or place of residence. Thus, the act of participating in public health measures (e.g., mask wearing or social distancing) was perceived as either a facilitator or mitigator of stigma, depending on one’s perceptions and identity (East Asian participants felt stigmatized for wearing masks, while Non-East Asian participants felt that those who did not wear masks or adhere to public health recommendations were stigmatized) [25]. Research suggests that members of the Chinese Canadian community living in Toronto closely followed the emergence of the coronavirus abroad, and many adapted public health measures — including masking, sanitization, and distancing — much earlier than others in the general public [1]. This theme was also noted by multiple participants in our study. Yet, rather than being seen as champions of preventive measures, participants in our sample felt they were stigmatized and, in some cases, verbally attacked for masking.

Our study purposefully recruited participants of Chinese ethnicity and was conducted in partnership with Chinese-Canadian advocacy organizations. While the intent of our study was to uncover perceptions of race-based stigma in Canada during COVID-19, we intentionally did not probe participants on their perceptions of stigma towards Chinese or East Asian individuals. Rather, we posed open-ended questions to allow participants to disclose their perceptions of who was being stigmatized in Canada during the pandemic and how. Interestingly, almost all participants in our study (including those of East Asian and non-East Asian ethnicity) described perceptions or experiences of race-based stigmatization towards East Asians. However, non-East Asian participants also described perceptions of other stigma marking towards other racialized groups, occupation, age, and place of residence. Many participants felt that the targets of COVID-19 related stigma changed as the pandemic evolved (e.g., first towards Chinese populations, then towards the US population or those out of province, then towards racialized, younger, and older adults in Ontario).

The impact of COVID-19 stigma marking was severe. An increase in reported racist incidents and hate crimes were noted in North America and spiked towards those of East and Southeast Asian ethnicity [3, 26–28]. In Canada, efforts by Dr. Teresa Tam on Twitter to address such racist remarks were met with vitriolic responses on Twitter to “stop race baiting” and “playing the race card” [29]. The majority of our participant sample were aware of racist or discriminatory attacks towards Chinese or East Asian individuals, and some directly experienced stigmatization, often in the form of unkind comments or unwanted looks in public spaces. Participants also described the negative impact of COVID-19 stigma on social inclusion, wellness, and mental health. Participants described not feeling comfortable navigating social spaces due to their race or ethnicity and fears of being stigmatized or discriminated against. Research shows that exposure to racist or stigmatizing media, even if not personally experienced, can contribute to increased risk of depression, anxiety, post-traumatic stress disorder, and mental unwellness [30, 31]. Fear of being stigmatized also affects health-seeking behaviors; for instance, research by Earnshaw et al. demonstrates that individuals who anticipated being stigmatized, and those who affirmed COVID-19 stereotypes, were less likely to get tested for COVID-19 [32].

Study participants believed that the media and the Canadian government did not do enough to stop or address COVID-19 stigmatization, discrimination, and anti-Asian rhetoric. Similar calls for action have been highlighted in academic literature, though there are limited data on the effectiveness of strategies to reduce stigma during public health emergencies [33–35]. A recent rapid review by Gronholm et al. provides guidance on potentially effective strategies [35] that should be considered by Canadian policymakers and media organizations. The authors suggest reducing stigmatizing language in media (e.g., presenting balanced views on disease burden, severity; not attaching locations to disease), developing anti-stigma campaigns to dispel misinformation and correct myths, and engaging with communities to build educational capacity and co-create empowerment strategies [35]. Such strategies should be rapidly developed, adapted iteratively in partnership with the impacted communities, and be tailored to population subgroups. Furthermore, there is a need for research that implements a social justice and equity lens. For instance, Mamuji et al. argue that it is not sufficient for research to stop at the reporting of stigma experiences, as this creates a “victimization narrative” of the impacted community [1]. Rather, understanding the deeper rooted, longstanding biases of a community can reduce perceptions of stigmatization as being a “temporary” phenomenon experienced during a health emergency (as perceived by some participants in our study) and may nudge communities to address these systemic biases.

Our findings align with other research describing experiences of discrimination and stigmatization among Asian-Canadian, Asian-Americans, and UK-Chinese populations amidst the COVID-19 pandemic [36–38]. A unique aspect of our study involves the inclusion of perspectives on COVID-19 stigma from non-East Asian individuals in Canada. Notably, and without prompting, these participants commonly reported witnessing or perceiving Chinese and East Asian individuals in Canada being stigmatized, discriminated against, or depicted negatively in the media. The saturation of these themes across participant group and across Western countries globally underscores the widespread prevalence of this phenomenon during the pandemic. Furthermore, we used the HSDF framework to systematically structure our thematic analysis (Fig. 1), which highlights key areas for research, intervention, monitoring, and policy as related to COVID-19 stigma, in particular towards Chinese and East Asian individuals.

This study has limitations. Recruitment of participants occurred primarily through online platforms, and as such, our sample may be missing perspectives of groups who are not active online. Additionally, most of our participants resided in urban cities in Ontario, which are culturally and racially diverse; this may have impacted the prevalence of stigma experienced and stigma perceptions. We did not collect data on place of birth or citizenship status, which may have impacted participants’ perceptions of stigmatization. Finally, we conducted these interviews between May and December 2020, which represented the first and second waves of the COVID-19 pandemic. As such, any changes in views and experiences due to subsequent waves of the pandemic are not represented in our results.

## Conclusions

In summary, participants in this study reported a number of stigma drivers, facilitators, and markers that contributed to their overall experiences with discrimination and stigmatization during the COVID-19 pandemic. These findings should be used to support the development of tools and strategies that can assist policymakers in addressing these stigmatizing factors as they emerge in real time during future disease outbreaks.

## Supplementary Information

**Additional file 1.** COREQ checklist.

**Additional file 2.** Interview Guide. Presents the interview guide rooted in the HSDF.

**Additional file 3.** Coding Framework. Presents parent, child nodes and descriptions used to code the interview data.

## Data Availability

The datasets generated and/or analyzed during the current study are not publicly available to protect the privacy of the participants and related confidentiality agreements. De-identified, available themed data can be made upon request to Christine Fahim (Christine.Fahim@unityhealth.to).

## Abbreviations

CCNCTO: Chinese Canadian National Council — Toronto Chapter
COREQ: Consolidated criteria for reporting qualitative studies
HSDF: Health Stigma and Discrimination Framework
PPE: Personal protective equipment
SARS: Severe acute respiratory syndrome
