# Supplementary material for: Exploring perceptions and experiences of stigma in Canada during the COVID-19 pandemic: a qualitative study
Source: BMC Glob Public Health. 2023 Dec 2;1:26. doi: 10.1186/s44263-023-00020-7 (PMC11116254; doi:10.1186/s44263-023-00020-7)
Supplement: Supplementary file 2 — Additional file 2. Interview Guide. Presents the interview guide rooted in the HSDF. [file 44263_2023_20_MOESM2_ESM.docx]

**Additional File 2: Public Member Interview Guide**

**Section 1: General Knowledge and Experiences**

1. **What are your thoughts on how the public has reacted to the COVID-19 outbreak?**
   1. Do you agree with what you have observed?

**Section 2: Drivers**

1. **How has COVID-19 impacted you personally?**
   1. How has it impacted your day-to-day life (e.g., job, health, family)?
2. **What is your greatest fear or concerns as related to COVID-19?**
   1. What are additional fears or concerns that you have?
3. **What are your thoughts on the financial impact COVID-19 will have on you or on others?**

Prior to this interview, we asked that you complete a demographic questionnaire. We believe in the importance of recognizing that individuals come from unique perspective that is shaped by their identity, such as their race, age, gender, and sexual orientation.

1. **Are there aspects of your identity (e.g., race, age, gender, sexual orientation) that has impacted your willingness to seek help if you thought that you were ill with COVID-19?**
   1. Do you think that there are certain individuals that due to their identity (e.g., race, age, gender, sexual orientation) would decline from seeking help if they thought that they were ill with COVID?
2. **Who would you feel comfortable reaching out to for help/support because of COVID-19 impacts?**

**Section 3: Facilitators**

1. **Are there any cultural, social, or gender norms that have influenced your views during the COVID-19 outbreak?**
2. **Do you think certain groups in the public may experience health consequences as a result of the COVID-19 outbreak compared to others?**
   1. If yes, please describe.

**Section 4: Stigma**

1. **Do you think fear and stigma around COVID-19 has resulted in certain groups being viewed by people in a negative way?**
   1. If yes, what aspects of someone’s identity, such as race, age, occupation, gender or sexual orientation, have been stigmatized because of COVID-19?
   2. What are your thoughts on the accuracy of that information?
   3. What are your feelings towards how certain groups are targeted?

**Section 5: Stigma Experiences**

1. **Have you experienced any stigma or discrimination because of COVID-19?**
   1. If you’re comfortable, could you share what that experience was like?
2. **Do you fear experiencing stigma because of COVID-19?**
