# Supplementary material for: Exploring perceptions and experiences of stigma in Canada during the COVID-19 pandemic: a qualitative study
Source: BMC Glob Public Health. 2023 Dec 2;1:26. doi: 10.1186/s44263-023-00020-7 (PMC11116254; doi:10.1186/s44263-023-00020-7)
Supplement: Supplementary file 3 — Additional file 3. Coding Framework. Presents parent, child nodes and descriptions used to code the interview data. [file 44263_2023_20_MOESM3_ESM.docx]

**Additional File 3 – Public Member Interview Stigma Coding Framework**

| Parent Node | Child Node | Node Description |
| --- | --- | --- |
| Perceptions of COVID-19 | 9.1 Participant's views and reactions | Participant's views and reactions to COVID-19 |
|  | 9.2 Family/friend's views and reactions | Family/friend's views and reactions to COVID-19 |
|  | 9.3 Public's views and reactions | Perception of public's views and reactions to COVID-19 |
| Impact of COVID-19 | 10.1 Personal impact | Impact of COVID-19 on personal daily life, including personal financial impact |
|  | 10.2 Economic activity | Impact of COVID-19 on indictors such as consumer prices, the unemployment rate, merchandise exports and imports, food supply chain, retail sales, hours worked and manufacturing sales, aircraft movements, and travel between Canada and other countries |
| Stigma drivers (factors that drive the stigmatization process) | 11.1 Fear of infection | Stigma associated with the fear of being infected/others infected with the virus |
|  | 11.2 Fear of social and economic ramifications | Stigma associated with the fear of social and economic ramifications |
|  | 11.3 Other fears | Stigma associated with the other fears |
|  | 11.4 Authoritarianism | Stigma associated with authoritarianism (power and authority) |
|  | 11.5 Lack of awareness | Stigma associated with the lack of knowledge about how COVID-19 spreads |
|  | 11.6 Social judgment | Stigma associated with social judgement (judgement by society) |
|  | 11.7 Stereotypes | Stigma associated with stereotypes (a fixed, over generalized belief about a particular group or class of people) |
|  | 11.8 Prejudice | Stigma associated with prejudice (preconceived opinion that is not based on reason or actual experience) |
|  | 11.9 Blame - misinformation | Stigma associated with the need to blame someone (for misinformation) |
|  | 11.10 Blame - COVID-19 outbreak | Stigma associated with the need to blame someone (for the outbreak) |
| Stigma facilitators (factors that influence the stigmatization process) | 12.1 Cultural norms | Stigma influenced by cultural norms (attitudes and patterns of behavior in a given group that are considered normal, typical or average within that group) |
|  | 12.2 Social and gender norms and equality | Stigma influenced by social and gender norms and equality |
|  | 12.3 Safety guidelines&standards (i.e., hand washing, social distancing) | Stigma influenced by safety guidelines&standards (i.e., hand washing, social distancing) implemented to reduce the spread of the outbreak |
|  | 12.4 Health policy | Stigma influenced by health policies to reduce the spread of the outbreak |
|  | 12.5 Media influence | Stigma influenced by media |
| Stigma marking (stigma applied to people of groups according to a specific health condition or other perceived difference such as race, class, gender, sexual orientation or occupation). | 13.1 Place of residence | Stigma applied to people due to their place of residence |
|  | 13.2 Race_ethnicity_culture_language | Stigma applied to people due to their race_ethnicity_culture_language |
|  | 13.3. Gender identity_sex | Stigma applied to people due to their gender or sexual orientation |
|  | 13.4 Education | Stigma applied to people due to their education |
|  | 13.5 Social capital | Stigma applied to people due to their social capital |
|  | 13.6 Occupation | Stigma applied to people due to their occupation |
|  | 13.7 Religion | Stigma applied to people due to their religion |
|  | 13.8 Beliefs | Stigma applied to people due to their beliefs |
|  | 13.9 Age | Stigma applied to people due to their age |
|  | 13.10 Socioeconomic status | Stigma applied to people due to their socioeconomic status |
| Stigma experience | 14.1 Discrimination | Discussions about discrimination experienced |
|  | 14.2 Stigmatization | Discussions about stigmatization experienced |
|  | 14.3 Fear of experience stigma | Discussions about anticipated fears of experiencing stigma (personally and for loved ones) |
| Support during COVID-19 | 15.1 Family/friends | Discussions on family/friends support due to the impacts of COVID-19 outbreak |
|  | 15.2 Health professionals | Discussions on health professional support due to the impacts of COVID-19 outbreak |
|  | 15.3 Others | Discussions on other examples of supports due to the impacts of COVID-19 outbreak |
